# Unveiling the potential anticancer activity of *Spirulina maxima* extract-nanoemulsion through in vitro and in vivo studies

**DOI:** 10.1038/s41598-024-82924-4

**Published:** 2025-01-06

**Authors:** Mohammed Yasser Hussein, Merna Nasr, Veronia Emad, Julie Maged, Portia George, Amina Emad, Abeer Mahmoud Badr, Mehrez E. El-Naggar, Sayeda M. Abdo, Jihan Hussein

**Affiliations:** 1 Biotechnology Program, Faculty of Science, Cairo University, Giza, 12612 Egypt; 2 Zoology Department, Faculty of Science, Cairo University, Giza, 12612 Egypt; 3 Textile Research Division, National Research Centre, Giza, 12622 Egypt; 4 Water Pollution Research Department, National Research Centre, Giza, 12622 Egypt; 5 Medical Biochemistry Department, National Research Centre, Giza, 12622 Egypt

**Keywords:** miR-221-3p, miR-222-3p, HepG2 cells, MCF-7 cells, Gallic acid, EAC model, Biochemistry, Biotechnology

## Abstract

Being the second leading cause of death globally, cancer has been a long-standing and rapidly evolving focus of biomedical research and practice in the world. Recently, there has been growing interest in cyanobacteria. This focus is particularly evident in developing innovative anticancer treatments to reduce reliance on traditional chemotherapy. This study investigates the anticancer potential of the *Spirulina maxima* extract nanoemulsion (SMNE) technique to improve the delivery, stability, and solubility of the *S. maxima* extract (SME). SMNE, prepared in three concentrations (SMNEC1, SMNEC2, SMNEC3), was characterized and confirmed to successfully load SME into silica-coated nanoparticles. Cytotoxicity tests on HepG2 and MCF-7 cell lines revealed a significant reduction in cell viability after 48-hour SMNE treatment, with IC50 values of 1488 µg/mL and 1721.936 µg/mL, respectively. SMNE also demonstrated efficacy in inhibiting tumor growth in mice with Ehrlich ascites carcinoma, normalizing alanine aminotransferase (ALT) and aspartate aminotransferase (AST) levels, and reducing oxidative stress markers such as catalase (CAT) and malondialdehyde (MDA). Histopathological examination showed that SMNEC3-treated groups had almost normal liver architecture. Additionally, SMNE downregulated oncogenic miR-221-3p and miR-222-3p, activating cancer suppression genes p27 and PTEN. The study concludes that SMNE, with its anti-inflammatory and antioxidant properties and ability to modulate key miRNAs, enhances SME delivery and shows promise as an effective cancer treatment.

## Introduction

Cancer is a significant global health threat, with rising incidence and mortality rates leading to millions of deaths each year for certain types of cancer^1^. The WHO has indeed reported projections of a significant increase in cancer cases and deaths by 2030, estimating 13 million cancer-related deaths and 21 million new cases annually^2^.

Various studies indicate that breast cancer and hepatocellular carcinomas are the most common types of cancer. According to 2024 WHO data, breast cancer is the most frequently diagnosed cancer among women, yet it ranks as the fifth leading cause of cancer-related deaths. In 2022, breast cancer resulted in approximately 670,000 deaths, highlighting its significant impact on mortality. Based on Global Cancer Observatory (GLOBOCAN) 2020 data, 2.3 million cases of breast cancer have been reported worldwide^3^. Additionally, liver cancer persists as a significant global health concern, with projections indicating an incidence of over one million cases by 2025 ^4^. It is the fourth most prevalent cause of cancer-related deaths worldwide and ranks fifth among the most common cancers. For liver cancer, hepatocellular carcinoma (HCC) is the most common kind^5^.

One of the most common therapies for cancer is chemotherapy. However, the existing chemotherapy techniques employed in clinical settings exhibit numerous drawbacks stemming from the limited precision of drug targeting and potent side effects^1^. These limitations substantially obstruct the efficiency of drugs, induce various dysfunctions within the body, and may even trigger the onset of additional illness. Despite their effectiveness, chemotherapy can lead to adverse reactions in healthy cells, manifesting as nausea, vomiting, mucositis, hair loss, neuropathy, and myelosuppression^6^.

Considering this perspective, natural products offer significant advantages characterized by their diverse chemical composition, minimal toxicity, safety profile, and accessibility. These attributes render them an appealing and cost-effective substitute for synthetic products^6^. A comprehensive investigation has been carried out on marine algae to examine its therapeutic and medical characteristics, such as its potential as an anticancer, antioxidant, and antibacterial agent^7^.

Among these marine organisms are cyanobacteria^8^, often known as blue-green microalgae, which are widely distributed prokaryotes capable of photosynthesis. They are regarded as highly effective sources of bioactive secondary metabolites. Commercial platforms cultivate more than 50% of cyanobacteria to extract bioactive compounds that have been shown to possess anticancer effects. Various natural compounds or their chemical counterparts can induce oxidative damage, affect mitochondrial function, cause cell cycle arrest, induce apoptosis, and alter cell signaling activation, all of which can result in cell death and potentially eliminate numerous cancer cells. Their therapeutic characteristics make them valuable for pharmaceutical and healthcare applications^9^.

Selecting *Spirulina* species among the current varieties of blue-green algae is advantageous due to their rich nutrient profile, containing numerous vitamins, minerals, phenolic acids, antioxidants, fatty acids, and bioactive compounds^10^. It is a filamentous cyanobacteria genus classified by botany specialists as a microalga belonging to Cyanophyceae^7^. Toxicological studies on all *Spirulina* species have not revealed any adverse effects on bodily functions during and after exposure to acute or chronic doses, which makes *Spirulina* spp. a preferable option compared to chemotherapy treatments^11^.

In our study, we worked on *S. maxima* because it offers many benefits, such as its quick cell development, abundant protein content, and crucial amino acid content, and it can also be easily scaled up^12^. *S. maxima*’s body surface is smooth and coverless; basic enzymatic systems may readily digest it. For this reason, we focused on the nanoemulsion formulation to protect and deliver our *S. maxima* extract (SME)^13^.

Nanoemulsion, in a definition, consists of nanoscale droplets with dimensions ranging from 10 to 200 nm, with each droplet coated with emulsifier molecules that provide protection^14^. Nanoemulsion remains a highly appropriate formulation choice despite the recent development of advanced nanostructures^15^. The key advantages of this formulation include excellent stability and solubility, which are necessary to produce an adequate therapeutic effect superficial charge, large surface area, elevated circulation half-life, particular targeting, and the formulation’s imaging capacity. Research on cancer therapy has shifted to a greater degree. The vascularized tissues surrounding cancer cells allow nanoemulsions to cluster easily and use their size to overcome barriers^16^. Most importantly, they can be developed to have specific targets, different types of drugs, and a purpose. They also can enhance the stability of chemicals by shielding them from unfavorable environmental conditions^17^. To increase our treatment’s efficiency and medical utilization, SME was loaded into the cavity of silica nanoemulsion (SNE), where silica has gained much interest in the nanomedicine field. Because of their biocompatibility and abundance in nature, The United States Food and Drug Administration has approved the use of silica as an adjuvant in the food industry^18,19^.

The objective of the designed work is to assess the anticancer potential effects of SMNE on breast cancer (MCF-7) and liver cancer (HepG2) cell lines. Additionally, the study aims to evaluate the biological changes in an in vivo model using mice bearing Ehrlich Ascites Carcinoma by evaluating liver enzymes (ALT and AST), oxidative stress biomarkers (CAT and MDA), histological examinations of liver tissue, and microRNAs (miR-221-3p and miR-222-3p) expression analysis through Quantitative Polymerase Chain Reaction (qPCR).

## Results

### Total phenolic content of processed SME

Table 1 demonstrates that the retention times of the sample align with the standard retention times for most of the compounds in the extract. According to the High-Performance Liquid Chromatography (HPLC) analysis, SME contains a mixture of significant phenolic compounds, including gallic acid (22.77 µg/g), methyl gallate (1.73 µg/g), and ferulic acid (0.37 µg/g).

**Table 1:** The concentration of phenolic compounds in the SME.

Peak	Phenolic contents	Retention time (min)	Area (mAU)	Conc. of phenolic compounds (µg/g)
1	Gallic acid	3.57	258.34	22.77
2	Chlorogenic acid	4.17	8.74	1.16
3	Catechin	4.29	20.52	4.59
4	Methyl gallate	5.28	33.57	1.73
5	Coffeic acid	5.78	26.21	2.08
6	Pyro catechol	6.52	9.30	1.38
7	Ellagic acid	7.18	3.79	0.64
8	Coumaric acid	8.28	9.54	0.39
9	Vanillin	8.92	9.77	0.35
10	Ferulic acid	9.56	1.65	0.37
11	Naringenin	10.77	5.51	0.10
12	Rosmarinic acid	11.78	10.87	0.52
13	Querectin	17.63	6.41	1.19
14	Cinnamic acid	19.81	6.75	1.24
15	Kaempferol	20.86	6.39	0.85
16	Hesperetin	21.21	11.49	0.12

### Total fatty acids content of processed SME

See Table 2.

**Table 2:** Fatty acid composition in SME using GC/MS.

Retention time (min)	Compound name	Molecular formula	Area %
14.07	Pentadecanoic acid	C15H30O2	0.15
16.9	Hexadecanoic acid, methyl ester Palmitic acid	C16H32O2	17
26.5	9,12-Octadecadienoic acid, methyl ester ‘linoleic acid’	C19H34O2	16.9
22.8	Octadecanoic acid ‘Stearic acid’	C18H36O2	0.1
29.6	Linolenic acid	C19H32O2	23.4
33.5	Docosadienoic acid	C22H40O2	0.1

Gas Chromatography/Mass Spectrometry (GC/MS) analysis identified six fatty acids in the SME, including three saturated fatty acids—pentadecanoic acid, palmitic acid, and stearic acid—and three polyunsaturated fatty acids—linoleic acid, linolenic acid, and docosadienoic acid, as detailed in Table. Linolenic acid was the most predominant fatty acid, accounting for 23.4% of the total area percentage.

### Characterization of SMEN

**Fig. 1:**
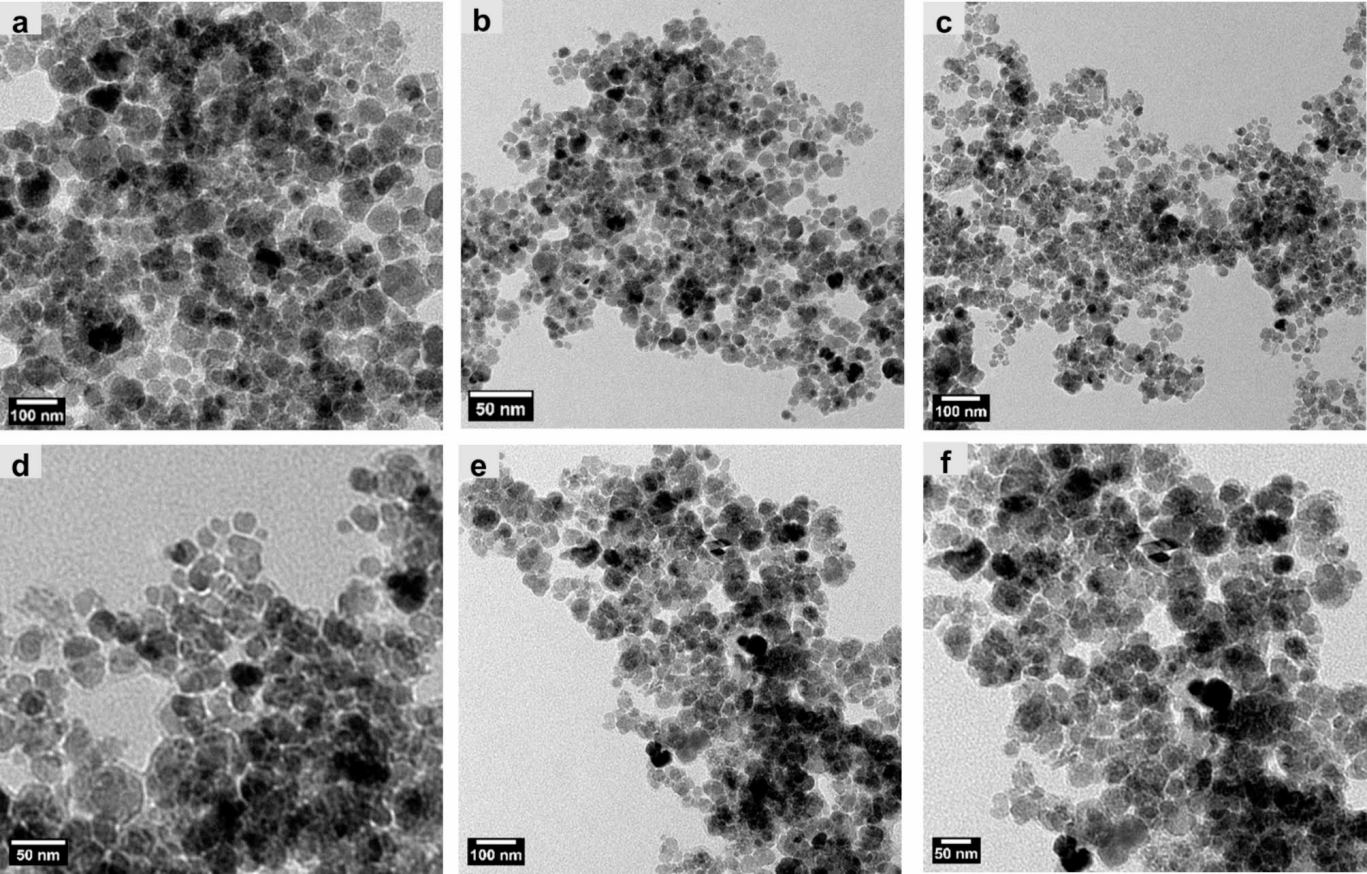
Transmission electron microscopy (TEM) images at two distinct magnifications of the three different concentrations of *S. maxima* extract nanoemulsion (SMNE): (**a**,**b**) represent concentration 1 (SMNEC1), (**c**,**d**) represent concentration 2 (SMNEC2), and (**e**,**f**) represent concentration 3 (SMNEC3) with respective concentrations of 3225 µg/mL, 6451 µg/mL, and 9677 µg/mL.

**Fig. 2:**
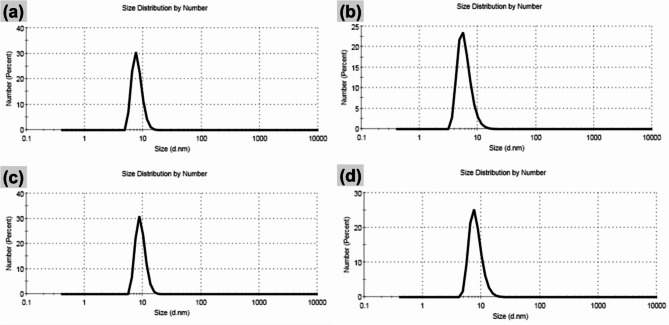
Average particle size of (**a**) silica nanoemulsion (SNE), and *S. maxima* extract nanoemulsion (SMNE) with (**b**) concentration 1 (SMNEC1), (**c**) concentration 2 (SMNEC2), and (**d**) concentration 3 (SMNEC3) with respective concentrations of 3225 µg/mL, 6451 µg/mL, and 9677 µg/mL.

**Fig. 3:**
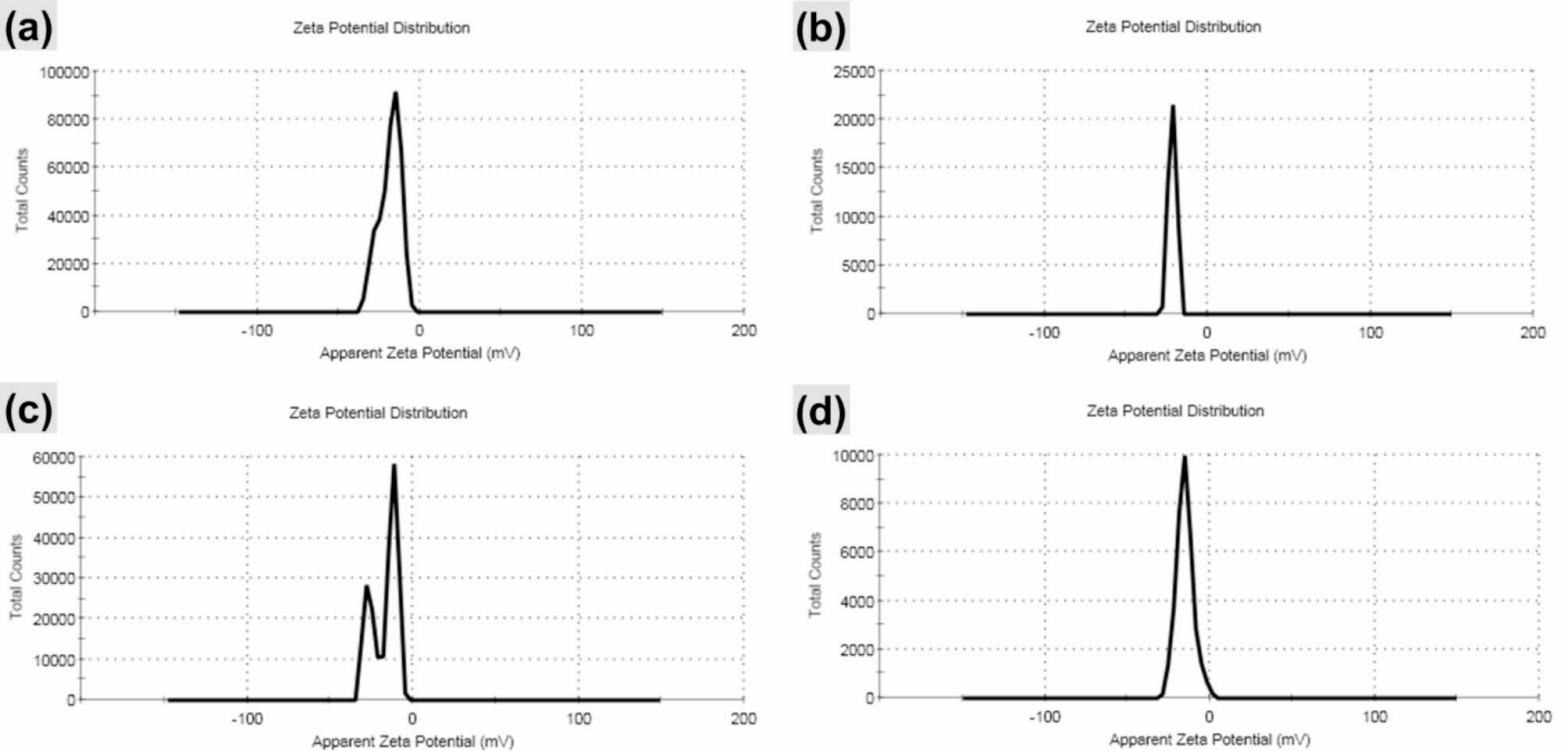
Zeta potential of (**a**) silica nanoemulsion (SNE), and *S. maxima* extract nanoemulsion (SMNE) with (**b**) concentration 1 (SMNEC1), (**c**) concentration 2 (SMNEC2), and (**d**) concentration 3 (SMNEC3) with respective concentrations of 3225 µg/mL, 6451 µg/mL, and 9677 µg/mL.

The morphology of the nanoemulsion was observed using TEM (Fig. 1). The particle size (Fig. 2) and zeta potential (Fig. 3) of the nanoemulsion were analyzed employing dynamic light scattering (DLS) using Malvern Zetasizer for different nanoemulsion samples. According to the literature, the SNE is prepared with a porous structure and formed as hollow spherical particles^20,21^. Visual inspection reveals the porous nature of the spherical particles, indicated by black regions, confirming the successful loading of SME into the hollow silica spheres (Fig. 1a–f).

The DLS of SNE (Fig. 2a) displayed that the particles are formed with a very small size (7.98 nm) with polydispersity index (PDI) = 0.361 and zeta potential of -18.4 Mv (Fig. 3a). The average particle size for SMNEC1 decreased to 6.054 nm (Fig. 2b) with PDI equal to 0.061, and for SMNEC2 and SMNEC3 increased to 8.199, 9.25 nm (Fig. 2c, d) with PDI equal to 0.669 and 0.645, respectively. Moreover, the averages of zeta potential were found to be -21.3, -16.9, and − 15 mV, as shown in (Fig. 3b,c,d) respectively.

### In vitro study

#### Cytotoxicity assay

**Fig. 4:**
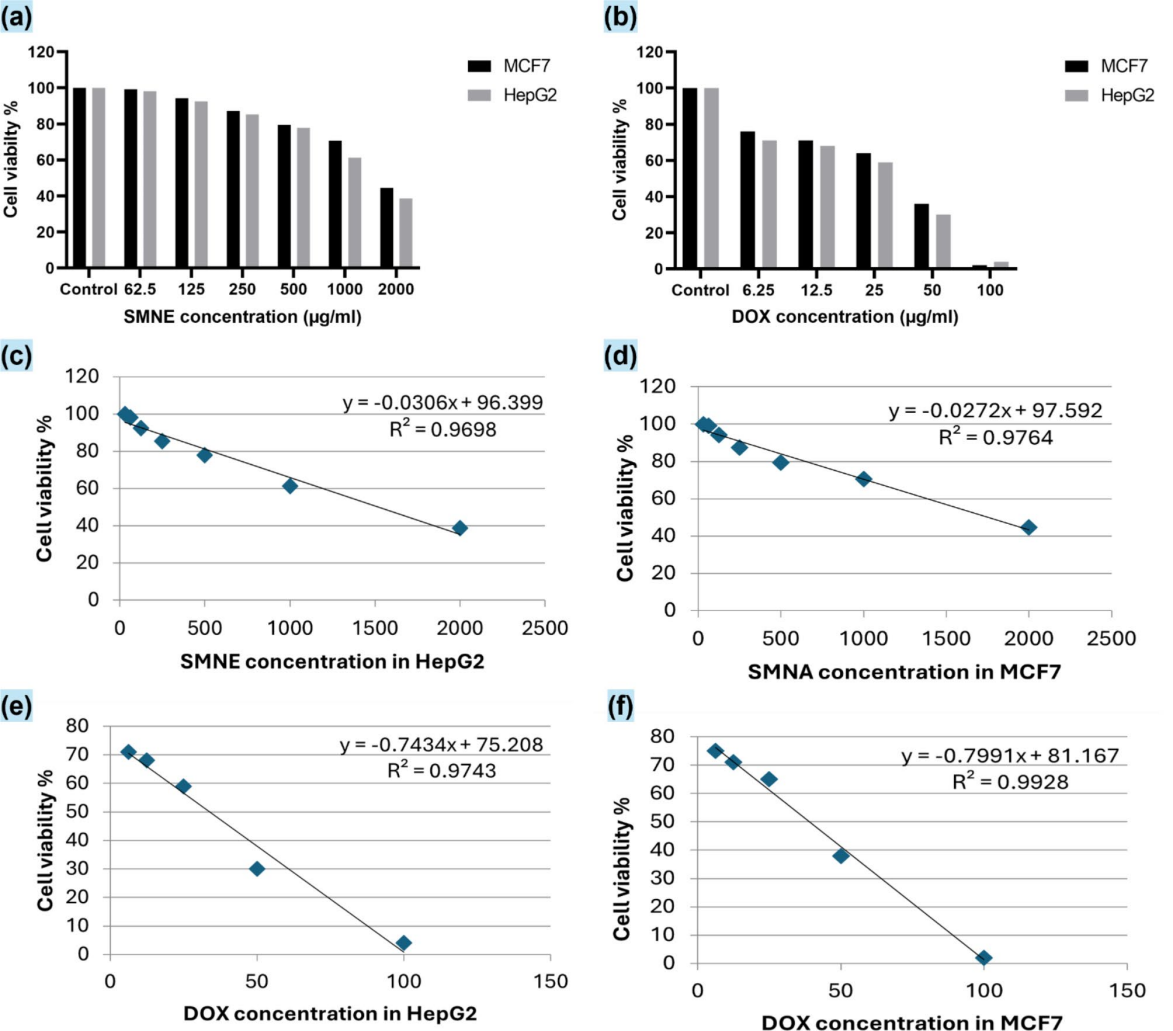
Effect of (**a**) *S. maxima* nanoemulsion (SMNE) and (**b**) Doxorubicin (DOX) on the viability of HepG2 and MCF-7 cell lines. The dose-response curves for SMNE on (**c**) HepG2 and (**d**) MCF-7 cells and for DOX on (**e**) HepG2 and (**f**) MCF-7 cells are shown.

To assess the cell toxicity of SMNE or DOX, an MTT assay was performed on HepG2 and MCF-7 cell lines, comparing treated cells to untreated controls. After 48 h, the MTT assay revealed that increasing doses of SMNE or DOX significantly reduced cell viability and induced cell death in a dose-dependent manner relative to the control (Fig. 4. a, b). The IC50 values for the SMNE were 1488 µg/ml for HepG2 cells and 1721.936 µg/ml for MCF-7 cells, whereas for DOX, the IC50 values were 34.03191 µg/ml for HepG2 cells and 38.50418 µg/ml for MCF-7 cells. These values were determined using linear regression analysis (Fig. 4. c, d, e, f).

### In vivo study

#### Mortality rate

According to Table 3, the SMNE treatment led to a reduction in the mortality rate compared to the EAC group. Specifically, the mortality rate in the EAC group was 62.5%, whereas SMNE treatment reduced this rate to 37.5% for SMNEC1 and SMNEC2, and 25% for SMNEC3.

**Table 3:** Mortality rate (%) post 14 days of treatment (day 7).

Groups	Day 7	Day 21	Dead no.	Mortality rate (%)
Normal	8	6	2	25
EAC	8	3	5	62.5
EAC + DOX	8	4	4	50
EAC + SME	8	4	4	50
EAC + SNE	8	4	4	50
EAC + SMNEC1	8	5	3	37.5
EAC + SMNEC2	8	5	3	37.5
EAC + SMNEC3	8	6	2	25

### Body weight changes

**Fig. 5:**
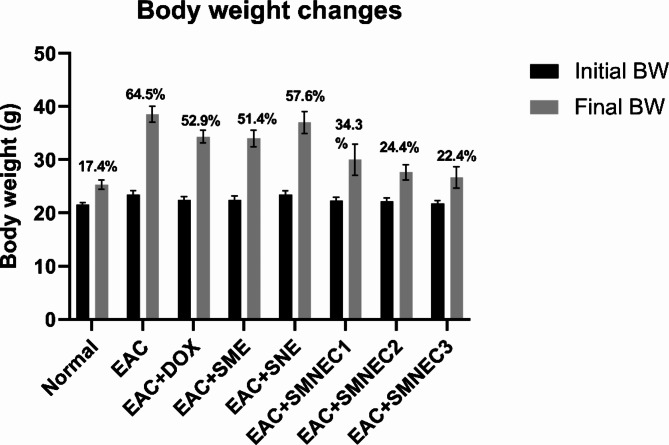
Initial and final body weights (BW) of mice subjected to various treatments. The values are expressed as mean ± standard error of the mean. The percentage values displayed above the gray bars represent the increase in body weight for each group. Normal: Mice without treatment; EAC: Ehrlich ascites-bearing mice without treatment; EAC + DOX: EAC-bearing mice treated with doxorubicin; EAC + SME: EAC-bearing mice with *S. maxima* extract treatment; EAC + SNE: EAC-bearing mice treated with SNE; EAC + SMNEC1: EAC-bearing mice treated with *S. maxima* extract nanoemulsion (SMNE) at 3225 µg/mL; EAC + SMNEC2: EAC-bearing mice treated with SMNE at 6451 µg/mL; EAC + SMNEC3: EAC-bearing mice treated with SMNE at 9677 µg/mL.

After 3 weeks from the start of the experiment, as illustrated in Fig. 5, the body weight change percent (BW %) in the normal control group was 17.4%. The EAC group exhibited the highest weight gain at 64.5% compared to all other treated EAC groups. while the EAC + SMNEC3 group had the lowest weight gain (22.4%) compared to all treated EAC groups. Specifically, the body weight percentages for the EAC-bearing mice treated with the three different concentrations of SMNE were 34.3% for SMNEC1, 24.4% for SMNEC2, and 22.4% for SMNEC3. In contrast, the EAC group treated with SNE showed minimal change in body weight% at 57.6%, similar to the untreated EAC group at 64.5%. Additionally, groups treated with DOX and SME showed comparable changes in body weight%, 52.9% and 51.4%, respectively.

### Ascites fluid volume

On the 22nd day of the experiment, changes in ascitic fluid volume were assessed. As detailed in Table 4, the groups treated with EAC + SMNEC1, EAC + SMNEC2, and EAC + SMNEC3 exhibited a significant reduction (*P* < 0.05) in tumor volume compared to the EAC group. In contrast, the EAC + SNE group showed no significant difference (*P* > 0.05) when compared to the EAC and EAC + SME groups. Additionally, there was no significant difference (*P* > 0.05) between the SME treatment group and the SMNEC1 treatment group.

**Table 4:** Total tumor volume in different groups under the study.

Groups	T.T.V. (mL/mouse)
EAC	16.33 ± 2.02^a^
EAC + DOX	4.66 ± 1.45^e^
EAC + SME	12.66 ± 2.33b^c^
EAC + SNE	14.67 ± 2.33^ab^
EAC + SMNEC1	5.33 ± 1.45^cd^
EAC + SMNEC2	5.33 ± 0.88^f^
EAC + SMNEC3	3.33 ± 0.88^g^

### Liver functions in mice

**Fig. 6:**
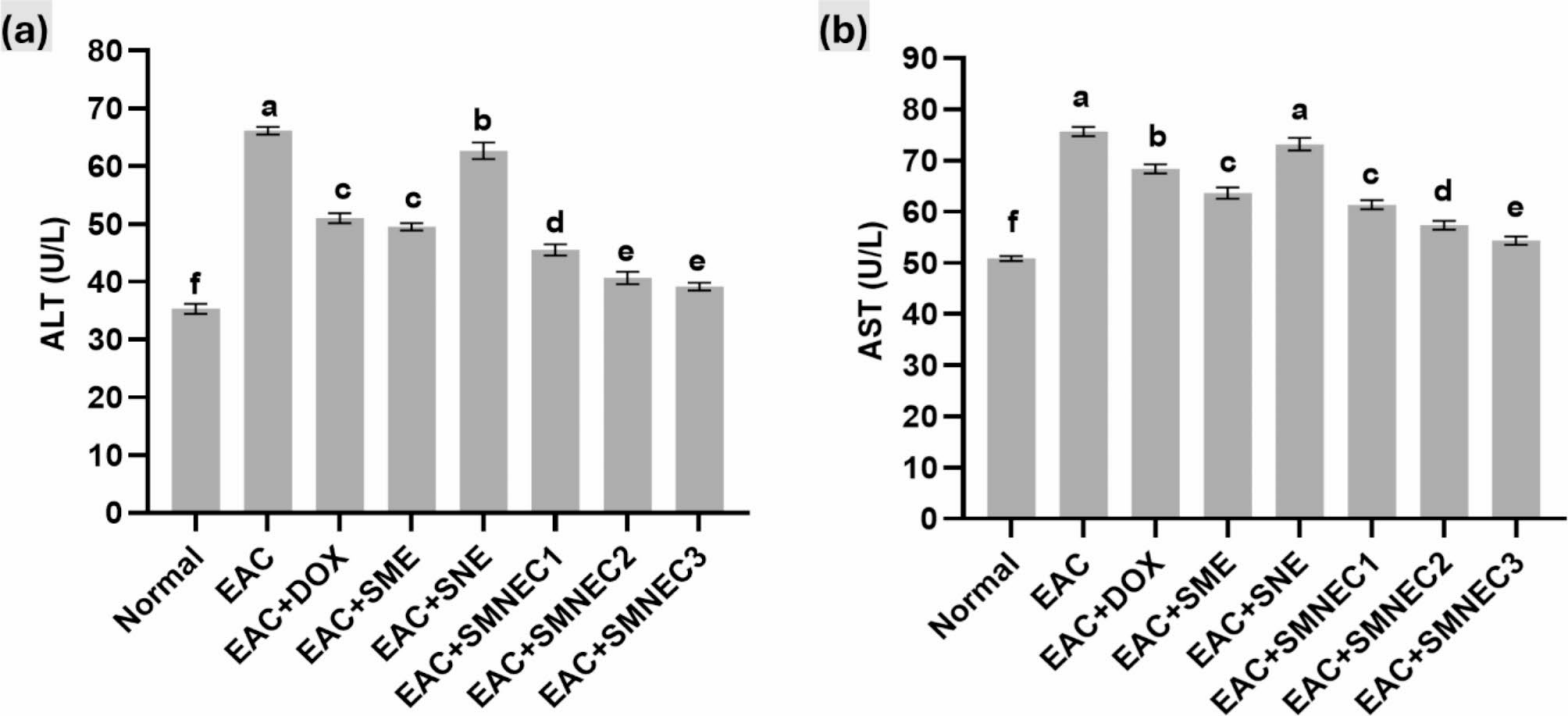
Serum levels of (**a**) ALT and (**b**) AST in all studied groups. Comparisons among groups were analyzed using one-way ANOVA followed by the least significant difference test. Values are presented as the mean ± standard error of the mean (*n* = 8). Different letters in the same histogram indicate significant differences (*p* < 0.05). While the same letters show non-significant differences (*p* > 0.05). Normal: Mice without treatment; EAC: Ehrlich ascites-bearing mice without treatment; EAC + DOX: EAC-bearing mice treated with doxorubicin; EAC + SME: EAC-bearing mice with *S. maxima* extract treatment; EAC + SNE: EAC-bearing mice treated with silica nanoemulsion (SNE); EAC + SMNEC1: EAC-bearing mice treated with *S. maxima* extract nanoemulsion (SMNE) at 3225 µg/mL; EAC + SMNEC2: EAC-bearing mice treated with SMNE at 6451 µg/mL; EAC + SMNEC3: EAC-bearing mice treated with SMNE at 9677 µg/mL.

As illustrated in Fig. 6, liver enzyme levels increased in the EAC group compared to the normal group. However, the treated groups (EAC + SMNEC1, EAC + SMNEC2, and EAC + SMNEC3) showed a significant decrease in AST and ALT levels compared to the EAC group (*p* < 0.05). The EAC + SME group showed no significant change in ALT activity compared to the EAC + DOX group (*p* > 0.05). The EAC + SMNEC2 and EAC + SMNEC3 groups displayed no significant difference from each other and had the lowest ALT activity compared to the normal group (*p* > 0.05). In terms of AST activity, the EAC + SNE group showed no significant change compared to the EAC group (*p* > 0.05), and the EAC + SME group showed no significant difference compared to the EAC + SMNEC1 group (*p* > 0.05).

**Fig. 7:**
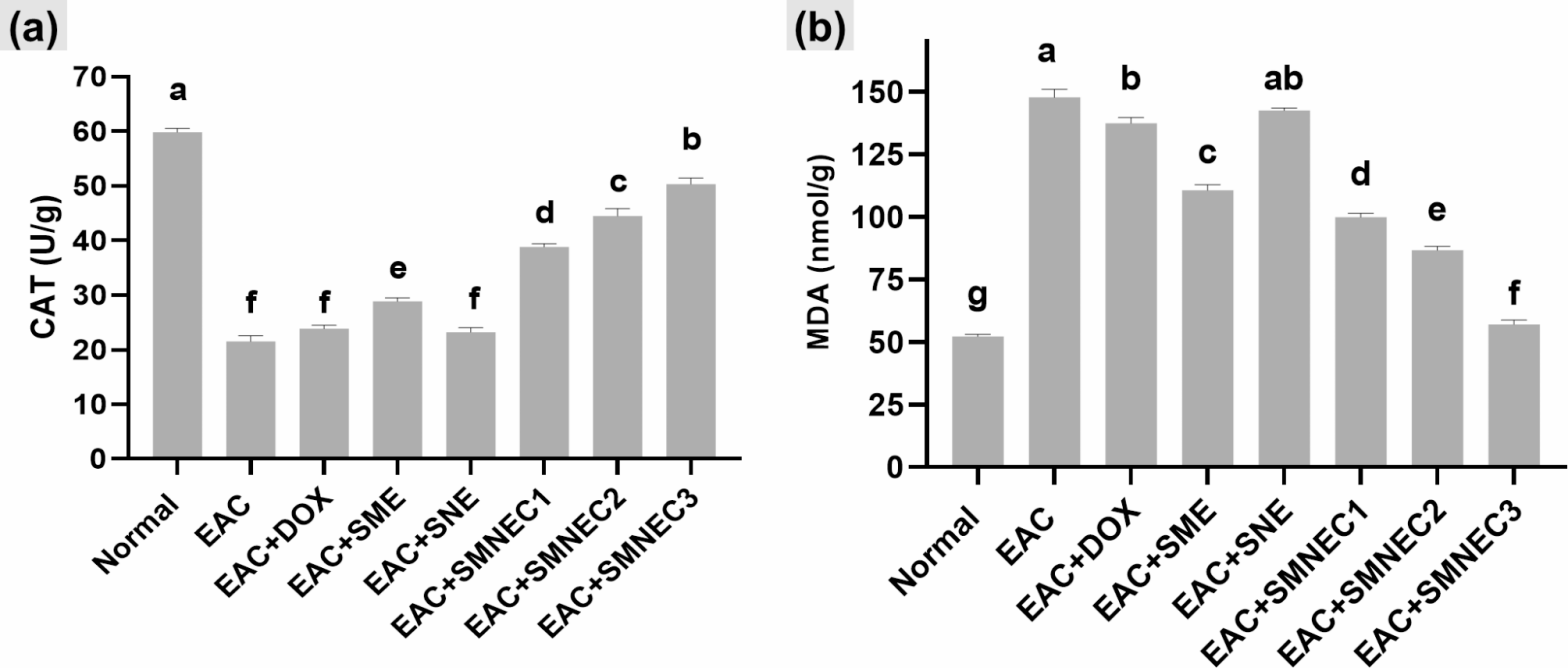
Liver tissue content of (**a**) catalase (CAT) and (**b**) malondialdehyde (MDA) activity in all studied groups. Comparisons among groups were analyzed using one-way ANOVA followed by a Least Significant Difference test. Values are presented as the mean ± standard error of the mean (*n* = 8). Different letters in the same histogram indicate significant differences (*p* < 0.05), whereas the same letters denote insignificance (*p* > 0.05). Normal: Mice without treatment; EAC: Ehrlich ascites-bearing mice without treatment; EAC + DOX: EAC-bearing mice treated with doxorubicin; EAC + SME: EAC-bearing mice with *S. maxima* extract treatment; EAC + SNE: EAC-bearing mice treated with silica nanoemulsion (SNE); EAC + SMNEC1: EAC-bearing mice treated with *S. maxima* extract nanoemulsion (SMNE) at 3225 µg/mL; EAC + SMNEC2: EAC-bearing mice treated with SMNE at 6451 µg/mL; EAC + SMNEC3: EAC-bearing mice treated with SMNE at 9677 µg/mL.

The results of the oxidative stress marker (CAT and MDA) are shown in Fig. 7. The control group has the highest CAT activity and lowest MDA levels, indicating effective antioxidant defense and minimal oxidative damage. In contrast, the DOX group shows reduced CAT activity and elevated MDA levels, suggesting compromised antioxidant defenses and increased cellular damage due to oxidative stress. Treatment with different concentrations of the SMNE (SMNEC1, SMNEC2, SMNEC3) results in varying degrees of CAT activity recovery and MDA reduction. The SMNEC3 group exhibits the most significant improvement in CAT activity and the lowest MDA levels among treated groups.

### MicroRNAs analysis

**Fig. 8:**
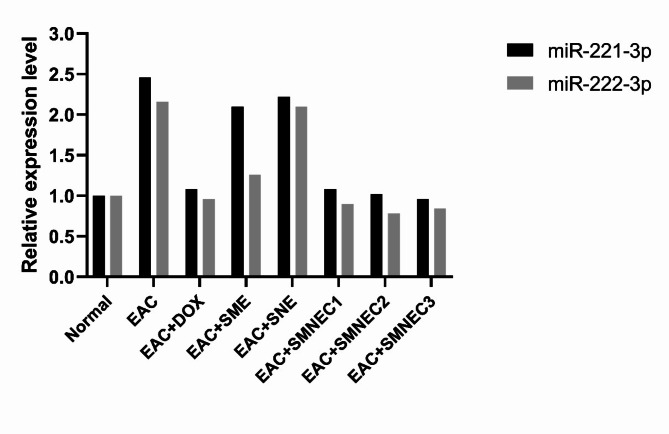
Relative expression level of miR-221-3p and miR-222-3p in liver tissues of all groups, measured by quantitative polymerase chain reaction (qPCR). Normal: Mice without treatment; EAC: Ehrlich ascites-bearing mice without treatment; EAC + DOX: EAC-bearing mice treated with doxorubicin; EAC + SME: EAC-bearing mice with *S. maxima* extract treatment; EAC + SNE: EAC-bearing mice treated with silica nanoemulsion (SNE); EAC + SMNEC1: EAC-bearing mice treated with *S. maxima* extract nanoemulsion (SMNE) at 3225 µg/mL; EAC + SMNEC2: EAC-bearing mice treated with SMNE at 6451 µg/mL; EAC + SMNEC3: EAC-bearing mice treated with SMNE at 9677 µg/mL.

For further investigation of the SMNE effect and sensitivity, the expression levels of oncogenes miR-221-3p and miR-222-3p were quantitatively assessed using qPCR (Fig. 8). The analysis revealed an increase in the fold change of both miR-221-3p and miR-222-3p in the EAC group compared to the control, indicating their role as oncogenes. Moreover, the EAC + SNE group showed no significant change in miR-221-3p and miR-222-3p levels compared to the EAC group, suggesting that SNE did not have a therapeutic effect. In contrast, the EAC + DOX group demonstrated downregulation of both miRNAs relative to the control. The EAC + SME group showed a minimal effect on miR-222-3p, which remained upregulated, while miR-221-3p exhibited no significant change. The SMNEC1, SMNEC2, and SMNEC3 groups showed similar effects on both miRNAs and were comparable to the DOX group, highlighting the enhanced sensitivity of this new formulation for the treatment compared to the extract alone.

### Liver histopathological examinations

See Fig. 9.

**Fig. 9:**
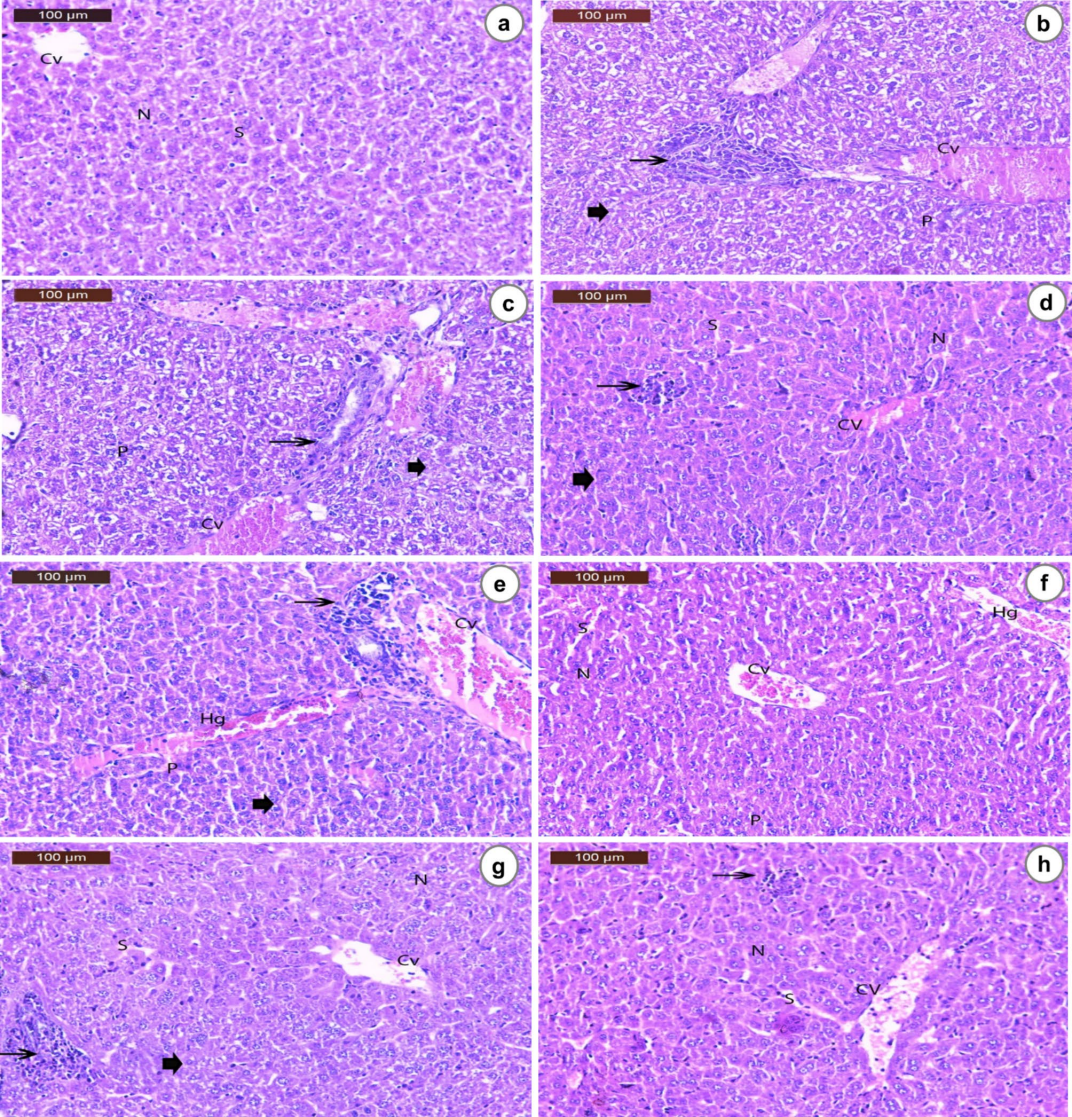
Photomicrographs of liver sections of mice stained with hematoxylin and eosin. (**a**) The Normal group exhibits normal hepatic architecture with hepatocytes radiating from the central vein (CV), blood sinusoids (S), and rounded vesicular nuclei (N). (**b**) The EAC group demonstrates a distorted normal architecture ranging from mild to moderate degree, with degeneration (arrowhead) and multiple patchy areas of inflammatory cell infiltrates, incomplete fibrous band formation, and connective tissue around the central vein (CV) and portal tract. Congestion of the central vein and pyknotic nuclei (P) were also observed. (**c**) The EAC + DOX group shows histopathological changes similar to the EAC group but with a higher degree of incomplete fibrous band formation and inflammatory cell infiltrates. (**d**) The EAC + SME group reveals liver architecture ranging from normal to mild degeneration (arrowhead), mild congestion of the central vein (CV), minimal inflammatory cell infiltrates (arrow), normal nuclei (N), and blood sinusoids (S). (**e**) The EAC + SNE group displays moderate histological and degeneration changes (arrowhead), noticeable congestion of the central vein (CV), inflammatory cell infiltrates (arrow), interstitial haemorrhage (Hg) and pyknotic nuclei (P). (**f**) The EAC + SMNEC1 group exhibits liver architecture ranging from normal to mild degeneration changes (arrowhead), mild congestion of the central vein (CV), mild interstitial haemorrhage (Hg), normal nuclei (N) and blood sinusoids (S). (**g**) The EAC + SMNEC2 group shows the nearly normal hepatic structure, with mild degeneration (arrowhead), central vein (CV), blood sinusoids (S), normal nuclei (N), and minimal inflammatory cell infiltrates (arrow). (**h**) The EAC + SMNEC3 group reveals an almost normal hepatic structure with hepatocytes radiating from the central vein (CV), blood sinusoids (S), normal nuclei (N), and a few numbers of inflammatory cells (arrow). Normal: Mice without treatment; EAC: Ehrlich ascites-bearing mice without treatment; EAC + DOX: EAC-bearing mice treated with doxorubicin; EAC + SME: EAC-bearing mice with *S. maxima* extract treatment; EAC + SNE: EAC-bearing mice treated with silica nanoemulsion (SNE); EAC + SMNEC1: EAC-bearing mice treated with *S. maxima* extract nanoemulsion (SMNE) at 3225 µg/mL; EAC + SMNEC2: EAC-bearing mice treated with SMNE at 6451 µg/mL; EAC + SMNEC3: EAC-bearing mice treated with SMNE at 9677 µg/mL.

### Graphical summary

See Fig. 10.

**Fig. 10:**
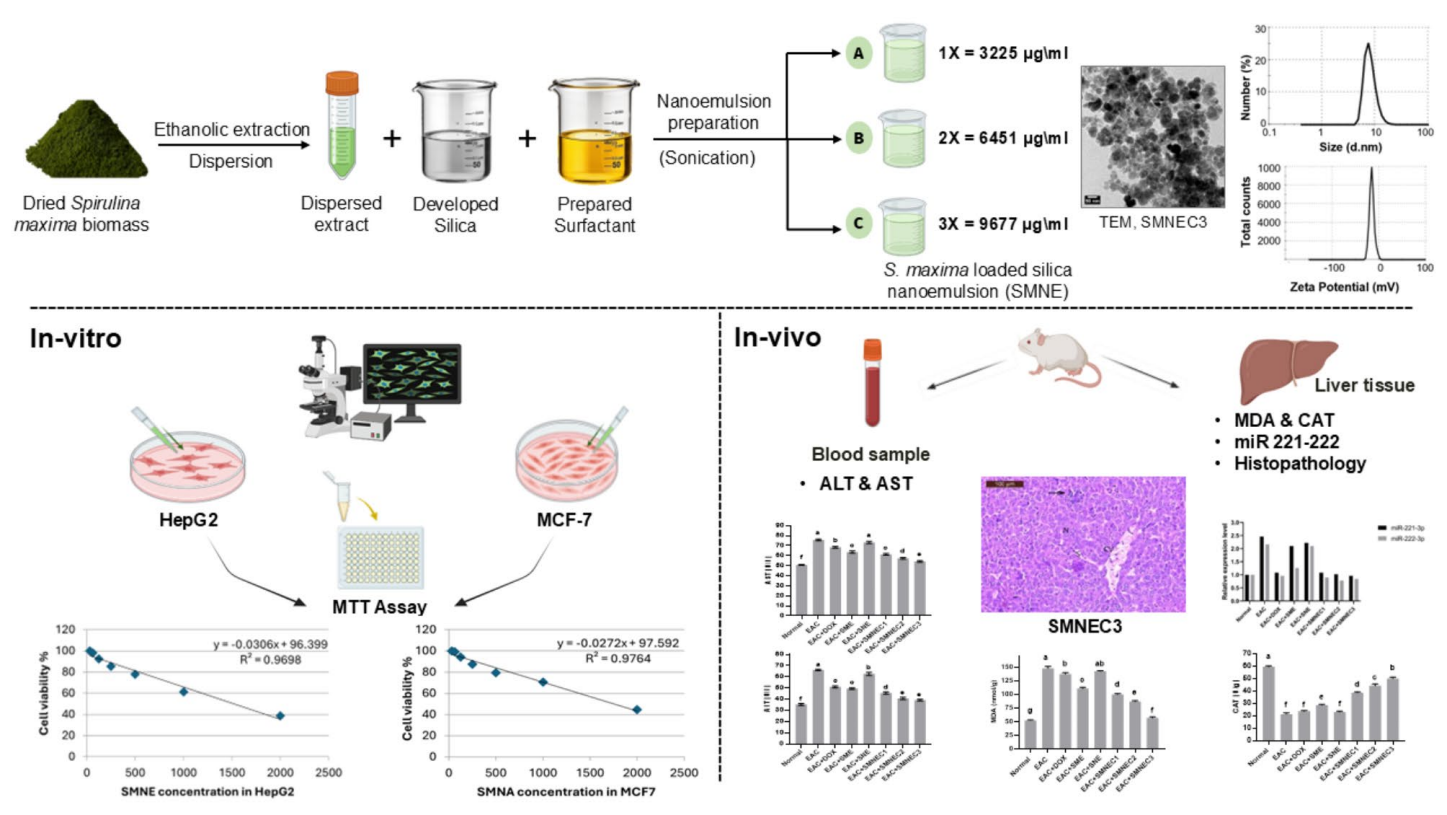
Graphical representation for the preparation and characterization of *Spirulina maxima* nanoemulsion (SMNE) with anticancer evaluation through in vitro (HepG2, MCF-7) and in vivo (biochemical, molecular, and histopathological) studies.

## Discussion

HPLC was employed to analyze the phenolic compounds in SME. The retention times of each component were compared to those of pure commercial standards for identification^22^.

Natural phenolic compounds are known for their anti-inflammatory and antioxidant properties, which play a crucial role in cancer prevention and treatment^23^. These compounds help regulate the reactive oxygen species levels and promote the expression of tumor suppressor proteins, such as p53. GA, the predominant phenolic compound in the extract (Table 1), is present at a concentration of 22.77 µg/g. This polyphenol exhibits various biological activities, including anti-inflammatory, antioxidant, antidiabetic, and antidepressant effects. Gallic acid also has potential therapeutic applications for oxidative stress-related disorders, including cancer. It influences cell signaling pathways in liver and breast cancers by regulating cell growth, survival, programmed cell death, angiogenesis, and reactive oxygen species imbalance^24^.

The fatty acid composition of SME was determined using GC/MS analysis (Table 2). Fatty acids are vital for numerous biological functions, serving as energy sources and membrane constituents. They also affect cell metabolism and responses to hormonal signals, and thus, play a role in various diseases, including metabolic disorders, inflammatory conditions, and cancer^25^. Linolenic acid is a carboxylic acid with 18 carbon atoms and three cis double bonds, existing as two isomers: α-linolenic acid and γ-linolenic acid^26^. The literature indicates that both isomers of linolenic acid exhibit anticancer effects against certain cancers, such as colorectal cancer^27^ and HCC^28^. This is achieved by inhibiting cell proliferation and promoting apoptosis. Additionally, another fatty acid present, palmitic acid (PA), has demonstrated anticancer activity^29^. PA, in particular, induces apoptosis through apoptosis-inducing factor (AIF) mediated cell death by depolarizing mitochondria and translocating AIF to the nucleus, thereby promoting cell death, especially in HCC^30^.

Nanoemulsions can concentrate in vascularized tissues, carry various medications, and target specific sites^17^. Silica oxide is the most prevalent inorganic substance utilized in biomedical nanocarriers. Porous silica prepared in nanoemulsions is frequently promoted as an effective method for drug loading and release^31,32^. SME loaded into SNE was achieved using sol-gel nanoemulsion employing TEOS as a precursor and Tween 80 as a surfactant and emulsifying agent^33^. Tween 80, also known as polysorbate 80, serves as both an emulsifying agent and a surfactant in the formation of SNE. As an emulsifying agent, it stabilizes emulsions by preventing the separation of immiscible substances. As a surfactant, Tween 80 reduces surface tension, preventing SNE particles from aggregating. Additionally, using an ultrasonic homogenizer aids in achieving complete dispersion for the formed nanoemulsion, ensuring no phase separation and enhancing the distribution of the particles throughout the emulsion. Furthermore, SNE was prepared with large pores capable of loading the *S. maxima* drug inside the silica cavities.

Nanoemulsion behavior may be influenced by a variety of physical and chemical factors. The morphology of the nanoemulsion indeed plays a crucial role in determining the future stability of the formulation. Optimizing the lipophilic–hydrophilic balance of the nanoemulsion is also crucial for drug loading and encapsulation efficiency^34^. Microscopic techniques have emerged as particularly valuable tools for obtaining reliable data about the morphology of the nanoemulsion system. TEM has been proven effective for investigating the internal structure of nanoemulsion droplets^17^. Moreover, the PDI measures the size distribution of nanoemulsion; the closer the PDI value is to 0, the more uniform the size distribution of the sample^35^. Surface charge, which affects stability and electrostatic interactions, is also important. Accurate measurement requires a magnetic field, as surface charge influences cellular response and is affected by the system’s size^36^.

It was also noted that the surfactant effectively envelops the SME particles, preventing them from clumping together. This observation confirms that the nonionic surfactant, Tween 80, plays a crucial role in stabilizing the nanoemulsion.

As known from the value of PDI, it can be concluded that the particles are formed with monodisperse particles with no noticeable agglomeration. The increase in particle size is primarily due to the swelling of the sample when it is dispersed in water. In addition, SMNE with high concentration (SMNEC3) is deposited onto the surface of silica nanoparticles, which, in turn, increases the whole particle size.

Overall, the PDI values indicate that the nanoemulsion has a consistent particle size distribution, which contributes to its long-term stability. Typically, an emulsifier helps reduce particle size by lowering the interfacial tension, which, in turn, increases the viscosity of the emulsion. This higher viscosity resists shear forces during nanodroplet formation^37,38^. When the volume of the aqueous phase is increased, while keeping the emulsifier amount and applied force constant, the energy is spread over a larger volume. This can reduce droplet breakdown, resulting in a higher PDI value. Additionally, differences in particle diameter measurements between TEM and DLS arise from the inherent variations between these techniques. Zeta potential, which reflects the surface charge of particles, can also indicate stability. Researcher suggests that even with a zeta potential less than ± 20 mV, as seen with the high-molecular-weight emulsifier Tween 80, adequate stability can be achieved due to steric hindrance, which aligns with our findings^39^.

The MTT assay results, shown in Fig. 4, reveal that SMNE effectively inhibits cell proliferation. Comparing the IC50 values for SMNE and the positive control, DOX, demonstrates that DOX exhibits greater anticancer efficacy at lower doses (IC50 = 34.03191 µg/ml for HepG2 and 38.50418 µg/ml for MCF-7) than SMNE (IC50 = 1488 µg/ml for HepG2 and 1721.936 µg/ml for MCF-7). This indicates that DOX is more potent than SMNE in this In vitro study. However, previous studies have highlighted several adverse effects of DOX, including cardiovascular issues^40^, allergic reactions, and bone marrow suppression^41^. In contrast, SMNE, with its natural composition, may offer a safer alternative. These findings will be further supported by in vivo investigations.

The mortality rate of EAC mice significantly increased, with a 62.5% rise compared to the normal group, likely due to the progression of untreated cancer. Notably, there was a significant reduction of 25% in mortality among EAC mice treated with SMNEC3. However, the mortality rate in DOX-treated EAC mice did not differ significantly from that in untreated EAC-bearing mice, or from the EAC + SME, and EAC + SNE groups, indicating that DOX treatment did not have a significant impact on mortality in this study (Table 3). These findings indicate that SMNE treatment is effective in extending the lifespan of the mice, providing evidence of its potential in cancer therapy and reducing mortality associated with the disease.

The inoculation of EAC cells resulted in an increased percentage of BW change, likely due to tumor progression caused by the proliferation of EAC cells within the peritoneal cavity. This increase in BW is further supported by the volume of ascitic fluid measured. Conversely, the percentage of BW change was reduced in groups treated with various concentrations of SMNE, indicating a potential tumor regression. This reduction in BW change is consistent with a decrease in ascitic fluid volume, reinforcing the efficacy of SMNE treatment in mitigating tumor growth. The results suggest that SMNEC treatments resulted in more controlled weight gain compared to the EAC and DOX groups.

On the 22nd day of the experiment, changes in ascitic fluid volume were assessed, as detailed in Table 4. Treatment of EAC-bearing mice with varying concentrations of SMNE (SMNEC1, SMNEC2, and SMNEC3) resulted in a significant reduction in total tumor volume (T.T.V.), demonstrating the effectiveness of the treatment compared to the SME and EAC-treated groups. This outcome indicates that the SMNE treatment effectively targets and reduces tumor progression. Since ascitic fluid serves as a critical nutrient source for EAC cell growth, a significant increase in its volume often signals tumor advancement^42^. The observed reduction in T.T.V. aligns with the body weight% results and is further supported by biochemical analyses and liver histopathological examinations.

ALT and AST are well-known blood-based circulating biomarkers that have long been used to assess liver damage. However, increased aminotransferases have been linked to human disorders and systemic regulation of metabolic activities^43^. The elevated levels of AST and ALT (Fig. 6) indicate hepatic disorder, corroborated by liver histopathological examinations. The progression of EAC-bearing mice led to hepatic damage, demonstrated by a significant increase in ALT activity. The rise in serum ALT and AST in EAC-bearing mice may be related to a disruption in transport function and enzyme leakage due to altered hepatic permeability^44^. However, the groups treated with the SMNE (EAC + SMNEC1, EAC + SMNEC2, and EAC + SMNEC3) showed near-normal levels of ALT and AST activity.

Oxidative stress has been investigated in various conditions, including kidney disease, liver cirrhosis, and cancer. Evaluating the redox status can assist in diagnosing and monitoring the progression of these illnesses. Measuring biomarkers such as CAT (Fig. 7a) and MDA (Fig. 7b) is essential for assessing the body’s redox state^45^. CAT is an essential antioxidant enzyme that protects cells by decomposing hydrogen peroxide, while MDA is a biomarker for oxidative stress and lipid peroxidation^46^. A negative correlation between CAT activity and MDA levels is observed, with increased oxidative stress leading to decreased CAT activity and elevated MDA levels. SMNEC3 highlighted its potential effectiveness in restoring antioxidant capacity and reducing oxidative damage. These findings suggest that oxidative stress exceeds the cellular antioxidant capacity, increasing lipid peroxidation. The nanoemulsion treatment effectively improves antioxidant defenses and mitigates oxidative damage, indicating its potential for therapeutic use in managing oxidative stress-related conditions.

The miRNAs miR-221-3p and miR-222-3p, when overexpressed, have been shown to directly downregulate the tumor suppressor and cell cycle regulator p27 (Kip1)^47^. Additionally, they specifically target Phosphatase and tensin homolog (PTEN) in breast cancer cells^48^ and in HCC^49^, inhibiting its activity. PTEN, the first tumor suppressor gene shown to have phosphatase activity, regulates various cellular activities, including cell cycle control^50^. These findings provide strong evidence of the oncogenic activity of miR-221-3p and miR-222-3p through their targeting of tumor suppressor genes.

Based on the gene expression analysis of the liver tissues in this study, the nanoemulsion significantly impacted gene expression sensitivity to treatment. Specifically, the three different concentrations of SMNE reduced the expression of both miRNAs. In contrast, SME showed reduced sensitivity by downregulating miR-222-3p, while miR-221-3p exhibited minimal alteration compared to the EAC group. This evidence confirms that the nanoemulsion composition enhanced the administration and effectiveness of the treatment.

Histopathological analysis of the EAC group (Fig. 9b) revealed distorted liver tissue architecture and the appearance of pyknotic nuclei, a hallmark of apoptosis^51^, indicating liver damage and inflammation. The liver sections from SNE-treated mice (Fig. 9e) also showed chronic inflammatory damage, marked by pyknotic nuclei, which correlated with elevated ALT and AST levels. This group did not exhibit any therapeutic effects from the cancer treatment, serving only as vehicle control for the extract delivery. In contrast, the SME group (Fig. 9d) demonstrated a similar and preferable effect on liver sections compared to the DOX (positive control) group (Fig. 9c). Treatment with SMNEC1, SMNEC2, and SMNEC3 showed significant improvements in liver structure. SMNEC1 (Fig. 9f) and SMNEC2 (Fig. 9g) restored nearly normal hepatic architecture (Fig. 9a), while SMNEC3 (Fig. 9h) exhibited slightly superior effects. Additionally, treatment with varying concentrations of SMNE enhanced liver architecture in the treated EAC groups, as evidenced by reduced ALT and AST serum levels.

## Materials and methods

### Materials

The dried algal biomass of *S. maxima* was supplied by the Hydrobiology Lab of the Water Pollution Research Department at the National Research Centre in Giza, Egypt. Tetraethyl orthosilicate (TEOS) and Tween 80 were acquired from Scientific Fischer Co. (USA). The cancer cell lines MCF-7 and HepG2 were purchased from the American Type Culture Collection (ATCC), located in Virginia, (USA). Tissue culture media was obtained from Invitrogen-Life Technologies. MTT was purchased from Acros Organics™ Thermo Fisher Scientific, USA. DOX was obtained from the drugstore in Egypt. Female CD1 Swiss albino mice were provided by the animal facility at the National Research Centr in Giza, Egypt.

### Preparation of SME

Dried algal biomass was immersed in 100% ethanol three times over three days. The mixture was then centrifuged for 10 min at 5000 rpm and 4 °C. The resulting supernatant was subsequently filtered through a filter paper (Whatman No. 1) to remove any residual cell debris^52^. After air-drying the samples, they were preserved at 4 °C until characterization and utilization.

### Preparation of SMEN

A dispersion of the extract with a concentration of 0.1 g/mL was prepared by adding 1 g of the dried extract to 10 mL of absolute ethanol and thoroughly mixed. A digital probe sonicator was used for 30 s for mixing 4 mL of TEOS with 20 mL of distilled water (dH_2_O). On the other hand, 2 mL of tween 80 was mixed with 5 mL of deionized water (diH_2_O) and placed in a digital probe sonicator for 2 min.

As graphically represented in Fig. 10, the nanoemulsion formulation involved adding three volumes of SME (1, 2, and 3 mL) dropwise to a Tween 80 solution, followed by homogenization to prepare three different concentrations of SMNE lapeled SMNEC1, SMNEC2 and SMNEC3, respectively. Taking into consideration, the total volume before and after extract addition was 31 mL. Subsequently, Tween 80 solution was added dropwise to the TEOS solution and the mixture was maintained under stirring for 30 min using a magnetic stirrer. Finally, the formed milky solution was further sonicated for 2 min using probe sonication. All these steps were performed to prepare the SNE without SME addition^53^. The resulting emulsions were kept at 4 °C till further characterization and utilization.

### Characterization

#### Estimation of the total phenolic content of processed SME

HPLC analysis of SME was performed utilizing an Agilent 1260 series system, with commercially available phenolic compounds serving as standards^22^. The separation was achieved using a Zorbax Eclipse Plus C8 column (4.6 mm x 250 mm ID, with 5 μm particles). The mobile phase consisted of water (A) and 0.05% trifluoroacetic acid in acetonitrile (B), and was pumped at a flow rate of 0.9 mL/min. The mobile phase was optimized using a linear gradient profile as follows: from 0 to1 min at 82% A, from 1 to 11 min at 75% A, from 11 to 18 min at 60% A, from 18 to 22 min at 82% A, and from 22 to 24 min at 82% A. A 5 µL injection volume was used for each sample, and the multi-wavelength detector was observed at 280 nm. The column temperature was maintained at 40 °C.

#### Estimation of total fatty acids content of processed SME

The lipid samples were subjected to methylation by heating with 5% methanolic HCL under reflux at 60 °C. Fatty acids were extracted using petroleum ether at temperatures between 40 °C and 60 °C. The ether extract was washed three times with distilled water, dried over anhydrous sodium sulfate, and then filtered^54^.

GC/MS analysis was conducted using a Thermo Scientific Trace GC Ultra/ISQ Single Quadrupole MS equipped with a TG5MS fused silica capillary column (30 m length, 0.251 mm diameter and 0.1 mm film thickness). The detection was carried out using an electron ionization device set to an ionization energy of 70 eV. Helium was used as the carrier gas at a constant flow rate of 1 mL/min. The injector and MS transfer line temperatures were maintained at 280 °C. The oven temperature was initially set to 150 °C and held for four minutes, followed by a programmed increase of 5 °C per minute until reaching 280 °C, where it was held for an additional four minutes. All detected components were quantified using the percent relative peak area. Preliminary identification of the compounds was performed based on comparative analysis.

The particle shape of SMNE was investigated using a JEOL 2010 F TEM at an accelerating voltage of 200 keV^55^. The average particle size of the nanoemulsion was determined through particle size analysis. Zeta potential analysis was performed to investigate the surface charges of the particles. Both particle size and zeta potential analyses were conducted with a Zetasizer Nano ZS dynamic light scattering equipment (Malvern Instruments, Malvern, UK)^56^. The samples were sonicated for 10 to 15 min before assessment.

### In vitro study

#### Human cancer cell lines and cell culture

A Laminar flow biosafety cabinet Class II A2 (Labconco) was utilized for all procedures performed in a sterile environment at 37 °C, while a CO_2_ incubator (Sartorius stedium, biotech) maintained the conditions. HepG2 and MCF-7 cells were cultured in Dulbecco’s Modified Eagle Medium (DMEM) with high glucose, 2 mM stable L-glutamine, 1% antibiotic-antimycotic mixture (containing 10,000 U/mL Potassium Penicillin, 10,000 µg/mL Streptomycin Sulfate and 25 µg/mL Amphotericin B), and 5% fetal bovine serum^57,58^.

#### MTT cytotoxicity assay

Cells were cultured in batches for 10 days, then plated at a density of 10,000 cells per well in fresh growth medium in 96-well plastic plates. They were incubated at 37 ºC for 24 h under 5% CO_2_, either alone (as a negative control) or with various concentrations of treatments (2000, 1000, 500, 250, 125, 62.5 µg/mL). After 48 h of incubation, the medium was removed, and 20 µl of MTT solution (2.5 µg/mL) was added to each well. The plates were incubated for an additional four hours at 37ºC under 5% CO_2_. To terminate the reaction and dissolve the formed crystals, 200 µL of 10% Sodium Dodecyl Sulfate in a 0.01 M HCL was added to each well, and the plates were incubated overnight at 37ºC. A positive control with 100 µg/mL, known to cause 100% lethality under the same conditions, was used to validate the cytotoxic^59^.

The absorbance was subsequently recorded with a microplate reader (Bio-Rad Laboratories Inc., model 3350, Hercules, California, USA) at 595 nm, using 620 nm as the reference wavelength. Cell viability and cytotoxicity were evaluated employing formulas (1) and (2):

1 $${\text{Viability }}\% =\left( {{\text{Test OD}}/{\text{Control OD}}} \right) \times {\text{1}}00$$

2 $${\text{Cytotoxicity }}\% ={\text{1}}00 - {\text{Viability }}\%$$

### In vivo study

#### Ehrlich ascites carcinoma cells

Ehrlich ascites carcinoma (EAC) cells were acquired from tumor-bearing mice obtained from the National Research Centre in Giza, Egypt. The live and dead cells were counted using the standard trypan blue exclusion method. The total number of viable EAC cells was determined by calculating the mean number of unstained cells and multiplying by 10^4 60^.

#### Mice and EAC cells inoculation

**Fig. 11:**
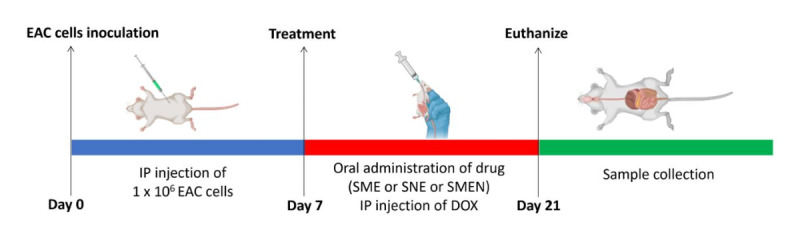
Timeline of conducting in vivo study.

Sixty-four female Swiss albino mice of the CD1 strain, weighing 20–25 g and approximately 5 weeks old, were obtained from Animal House Colony at the National Research Center in Giza, Egypt. After a seven-day acclimatization period at the Animal House Lab in NRC the mice received an intraperitoneal (IP) injection of 1 × 10^6^ EAC cells. Then, they were allowed to undergo tumor progression for an additional seven days before the commencement of treatment (Fig. 11). During this period, the mice were fed a standard lab diet.

All experimental protocols were reviewed and approved by the Animal Care and Use Committee of the Faculty of Science, Cairo University, Giza, Egypt (Approval Number: CUIF7223). The study was conducted in strict accordance with the relevant guidelines and regulations and adhered to the ARRIVE guidelines for the ethical treatment of animals^61^.

### Experimental design

**Fig. 12:**
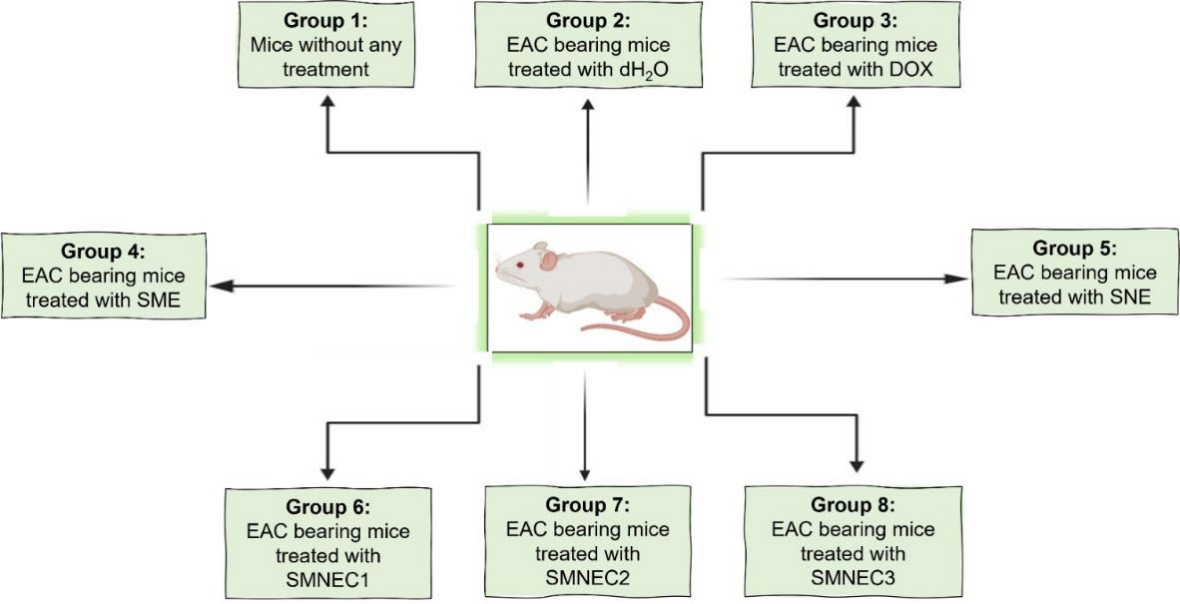
Schematic presentation of mice groups of the in vivo study.

The sixty-four mice were randomly assigned to eight groups, as illustrated in (Fig. 12), with eight mice per group. The groups were classified according to the specific treatment they received. Group 1 represented untreated normal mice. Group 2 consisted of mice with EAC cells exposed to dH_2_O as a stress-inducing agent. Group 3 comprised mice with EAC cells administered DOX as a positive control for the treatment. Group 4 involved mice with EAC cells treated with an SME. Group 5 consisted of mice with EAC cells given SNE as a vehicle control for the treatment. Groups 6, 7, and 8 received varying concentrations (3225, 6451, and 9677 µg/mL) of SMNE (SMNEC1, SMNEC2, SMNEC3), respectively. The in vivo LD50 of acute toxicity was estimated from in vitro IC50 ^62,63^ using the following formula (3):

3 $${\text{log LD5}}0=0.{\text{372 }} \times {\text{ log IC5}}0{\text{ }}({\text{mg}}/{\text{mL}})+{\text{2}}0.0{\text{24}}$$

SMNE treatments were administered orally daily for 14 days at a dosage of 1/20th of the LD50, while DOX was IP injected at a dosage of 1/10th of the LD50.

### Mortality rate estimation

Mice were observed for mortality recording throughout the experiment. From this observations, the percentage (%) of mortality for each group was calculated using the following formula^64^ (4):

4 $$\% {\text{ mortality}}={{\text{N}}_{\text{t}}}/{{\text{N}}_0} \times {\text{1}}00$$

where N_t_ is the number of dead mice in each group, and N_0_ is the number of mice taken in each group for testing (i.e., 8).

### The body weight changes of the mice

The weights of the mice were recorded at the start of the experiment (referred to as the initial body weight) and at the end (referred to as the final body weight). The formula (5) used to compute the percentage change in body weight (% BW) was as follows^65^ :

5 $$\% {\text{ BW}}=\left( {{\text{final BW}} - {\text{initial BW}}/{\text{initial BW}}} \right) \times {\text{1}}00$$

### Samples collection

Twenty-four hours after the final dosage (day 21), the mice anesthetized with 2% isoflurane (primary method). Blood samples were collected from each group via the retro-orbital plexus, then centrifuged at 5000 rpm for 5 min. The serum was then separated and stored at -80 °C for further analysis.

The cervical dislocation (secondary method) was performed for the euthanasia procedure. Meticulous dissection was used to carefully collect ascitic fluid from groups 2–8. The volume of ascetic fluid was measured using needle aspiration (18–22 gauge). Liver tissues from all groups were collected and divided into two sets: one set was preserved in 10% formalin for histopathological analysis, while the other was stored at -80 °C for miRNA qPCR and other analytical processes.

### Liver histological examinations

The liver tissues were preserved in 10% formalin saline, sectioned at 5 μm thickness using a microtome, and subsequently embedded in paraffin blocks. The sections were stained with hematoxylin and eosin for histopathological analysis and were examined under a light microscope^66,67^.

### Biochemical analysis

According to the manufacturer’s instructions, total RNA was isolated from liver tissues using QIAzol^®^ lysis reagent (Qiagen, Hilden, Germany). The isolated RNA was reverse transcribed into cDNA using the SuperScript™ III cDNA synthesis kit (Thermo Fisher Scientific, Inc., Waltham, MA, USA). The Tagman™ MicroRNA Assay (cat. no. 4427975; Thermo Fisher Scientific, Inc., Waltham, MA, USA) was included in this reaction to synthesize cDNA for specific miRNA (miR-221-3p, ID: 000524; miR-222-3p, ID: 002276; and U6, ID: 001973).

The reverse transcription PCR was conducted with the following conditions: 25˚C for priming for 10 min, 46˚C for reverse transcription (RT) for 20 min, and 95˚C for RT inactivation for 1 min. Subsequently, quantitative real-time PCR was performed in a PCR mixture using the 20X TaqMan miRNA Asaasy and 2X TaqMan™ Universal PCR Master Mix, following the manufacturer’s instructions. The RT-qPCR conditions were as follows: 40 cycles at 95˚C for 15 s and 60˚C for 1 min. Data were analyzed using the comparative threshold cycle value (2^−ΔΔCt^) method^67^.

Liver function tests were performed to measure the levels of liver enzymes, specifically ALT and AST, following the method described by Reitman and Frankel^68^. Diagnostic kits from Roche Diagnostics Ltd (Germany) were used for these tests.

Liver MDA activity, an indicator of oxidative stress, was measured using the methodology described by Uchiyamara and Mihara^69^. Additionally, the activity of CAT, an antioxidant enzyme, was assessed using a standard spectrophotometric assay method developed by Claiborne^70^.

### Statistical analysis

All the statistical analyses were conducted using IBM SPSS Statistics for Windows, Version 26.0 (IBM Corp., 2019, Armonk, NY) and GraphPad Prism for Windows, Version 9 (GraphPad Software, 2020, La Jolla, CA), with results reported as means ± standard error of the mean. One-way analysis of variance (ANOVA) was used and a probability value of 0.05 or less was considered statistically significant.

## Data Availability

Available under request from the corresponding author. MohammedYMMH@gmail.com.

## Abbreviations

SME: Spirulina maxima extract
SNE: Silica nanoemulsion
SMNE: Spirulina maxima extract nanoemulsion
DOX: Doxorubicin
TEOS: Tetraethyl ortho silicate
TEM: Transmission electron microscope
PDI: Polydispersity Index
DLS: Dynamic light scattering
rpm: Revolution per minute
HCC: Hepatocellular Carcinoma
AIF: Apoptosis-inducing factor
EAC: Ehrlich ascites carcinoma
RT: Reverse transcription
qPCR: Quantitative polymerase chain reaction
HPLC: High-performance liquid chromatography
MTT: 3-[4,5,dimethylthiazol-2-yl]-2,5,diphenyl tetrazolium bromide
IC50: Inhibitory concentration 50
ATCC: American type culture collection
DMEM: Dulbecco’s modified eagle medium
ALT: Alanine aminotransferase
AST: Aspartate aminotransferase
MDA: Malondialdehyde
CAT: Catalase
IP: Intraperitoneal
CV: Central vein
S: Blood sinusoids
N: Nucleus
T.T.V: Tumor total volume
BW: Body weight
ANOVA: Analysis of variance
